# Case Report: External fixation of airway stents for management of tracheal stenosis secondary to thyroid cancer

**DOI:** 10.3389/fsurg.2026.1801805

**Published:** 2026-06-03

**Authors:** Conghui Liu, Ziyang Zeng, Ziyan Wang, Xinran Li, Jingpu Hou, Aishuang Fu, Yanlei Ge

**Affiliations:** 1 Department of Endocrinology, North China University of Science and Technology Affiliated Hospital, Tangshan, Hebei, China; 2 Department of Respiratory Medicine, North China University of Science and Technology Affiliated Hospital, Tangshan, Hebei, China; 3 Department of Thoracic Oncology Surgery, North China University of Science and Technology Affiliated Hospital, Tangshan, Hebei, China

**Keywords:** airway stents, bronchoscopic intervention, stent fixation, thyroid cancer, tracheal stenosis

## Abstract

**Background:**

Thyroid cancer, in the advanced stage, often invades the airway and leads to tracheal stenosis. Airway stents can be used as a palliative treatment to restore airway and relieve dyspnea. However, stent migration remains a frequent complication, especially for lesions near the glottis. Simple fixing technology suitable for environments with limited resources is rarely reported.

**Methods:**

A 79-year-old woman with advanced thyroid cancer developed upper tracheal stenosis due to tumor compression. To restore airway patency, the patient accepted placement of a self-expanding metallic stent in the upper airway, but early migration occurred. Under combined ultrasound and bronchoscopic guidance, a simplified external fixation technique using a button was performed.

**Results:**

The stent restored tracheal patency, and the stent position was stabilized following external fixation. During the short-term follow-up, the stent remained stable, but the tumor progression, granulation tissue formation, and secretion retention were observed.

**Conclusions:**

The technique of external fixation with an airway stent offers a new choice for subglottic airway stenosis caused by thyroid cancer in elderly patients. This technology helps maintain the stent position in the short term in an environment with limited medical materials. However, further observation and a longer follow-up period are needed to assess its generalizability and safety.

## Introduction

Airway invasion is a life-threatening complication of advanced thyroid cancer. As the disease progresses, the tumor can cause compression, deformation, and narrowing of the trachea, leading to difficulty in breathing (1). Traditional treatment regimens such as surgical resection, radioactive iodine-131 therapy, radiotherapy, and chemotherapy often exhibit delayed efficacy or significant adverse effects. Additionally, interventions such as tracheal intubation and surgery are often limited by the patient's physical condition (2, 3). The integration of interventional methodologies with airway stent placement has established itself as a pivotal approach for the prompt mitigation of respiratory distress induced by airway stenosis (4–6). Airway stents generally include silicone and metallic types, which differ in their performance in terms of flexibility, radial strength, and procedural convenience (7–9). However, regardless of the type of stent, stent migration is one of the most common and challenging complications in clinical practice, especially in the upper tracheal stenosis near the glottis. Several fixation methods have been proposed to address this problem, but most rely on specialized instruments or complex procedures, limiting their application in environments with limited equipment or resources (10–12). We describe a patient with upper tracheal stenosis caused by thyroid cancer invasion who developed early stent migration and was subsequently managed using a simplified external fixation technique based on an ordinary button. This study aims to explore the feasibility and clinical practical value of this method under the condition of limited resources.

## Case and methods

A 79-year-old female patient presented to our hospital on april 6, 2024, with a cough and shortness of breath. The patient had been diagnosed with thyroid cancer one year prior and had not undergone thyroidectomy due to advanced age and refusal of surgery. Histopathological findings at that time suggested squamous differentiation. Bronchoscopy revealed that the tumor had begun to invade the airway, causing compression and narrowing. The tumor stage was clinical stage (c)T4aNxM0. The tumor initially infiltrated the airway, accompanied by hoarseness, choking on drinking or coughing, and intermittent dyspnea. Bronchoscopic examination revealed that the airway was approximately 70% obstructed. Because the patient firmly refused thyroidectomy, we performed cryotherapy using carbon dioxide as a refrigerant on the mass under bronchoscopic guidance and used biopsy forceps to remove part of the mass, thereby alleviating the patient's dyspnea. The patient was discharged after postoperative relief of dyspnea symptoms. After discharge, the patient received regular targeted therapy with dabrafenib 150 mg 2/day combined with trametinib 2 mg/day. During this period, the patient did not undergo regular follow-up examinations, and no significant drug-related adverse reactions were observed. At this admission, the patient exhibited marked dyspnea at rest compared to previous episodes, accompanied by coughing and wheezing. During physical examination, the patient's bilateral thyroid gland had multiple palpable lumps with ill-defined boundaries, smooth surface, no tenderness, and were mobile with swallowing. Initially, it was considered that the patient's shortness of breath was caused by progressive tracheal stenosis due to tumor invasion. After admission, the patient was given methylprednisolone 2 mg/day combined with salbutamol 0.63 mg 2/day by nebulization to dilate the airways. The patient had a cough and sputum, and was given protease 4,000 u three times a day to help expectoration. Noninvasive ventilation was initiated to provide ventilatory support due to progressive dyspnea. On april 7, 2024, the chest ct revealed upper tracheal stenosis and leftward tracheal deviation (Figure 1A). On april 8, 2024, bronchoscopy revealed that the stenosis was located in the upper trachea, approximately 3 cm below the glottis, where the airway was compressed by a thyroid cancer mass, resulting in nearly 80% luminal obstruction (Figure 2A). According to the myers-cotton classification of subglottic stenosis, the patient was diagnosed with grade iii tracheal stenosis. Due to severe extrinsic compression causing critical airway stenosis, bronchoscopic cryotherapy combined with tumor debulking using biopsy forceps was performed, partially restoring airway patency (Figure 2B). With the assistance of balloon dilation, a 16 mm diameter, 5 cm long straight self-expanding metallic stent was placed in the patient's upper trachea. Postoperatively, the patient's dyspnea improved compared to before. However, one day after surgery, the patient developed severe coughing accompanied by marked wheezing and difficulty expectorating sputum. Bronchoscopic images revealed that the stent had slipped from its original position to a location below the stenosis. To ensure effective airway stent support and prevent airway collapse, after the stent was readjusted to the narrowest part of the upper trachea, the external fixation of the airway stent was performed by the double-needle method. First, under the guidance of ultrasound, the pre-installed 3-0 non-absorbable suture was used to puncture the cricothyroid membrane and penetrate into the tracheal cavity. Under bronchoscopic visualization, the suture ends were externalized and secured extracorporeally using a standard surgical button placed on the anterior neck skin. The button served to distribute tension and maintain stable fixation of the stent. The external button device was used to reinforce fixation and prevent stent migration (Figure 3). Postoperative evaluation showed good stent patency and airway support, and no active bleeding. Airway secretions were adequately cleared. On april 15, 2024, an electronic bronchoscopy showed that the stent was well-positioned. The patient's shortness of breath has improved, discharged 8 days after airway stent implantation. During short-term follow-up, the patient was admitted on may 22, 2024, for an examination of the airway stent status. The patient experienced wheezing, accompanied by cough and sputum production. Bronchoscopy revealed the tracheal stent was in good position, and purulent sputum was observed within the lumen and on the surface of the stent. After sputum removal under bronchoscopy, the patient's dyspnea was relieved. Unfortunately, two months after the patient's airway stent was placed, the patient was hospitalized again due to difficulty breathing. The neck was obviously swollen, with no redness, swelling, ulceration, or infection at the suture site. In june 2024， bronchoscopy observed that there was no migration of the airway stent; however, tumor infiltration was noted from the upper edge of the glottis to the stent, and granulation tissue was observed at the lower edge of the stent (Figure 4). Chest ct revealed further infiltration of the thyroid tumor (Figure 1B). The rapid progression of thyroid cancer compared with the previous condition made the patient unable to cough effectively and expectorate sputum, resulting in airway obstruction due to retained secretions. After the last treatment session, the patient requested discharge against medical advice and was ultimately lost to follow-up.

**Figure 1:**
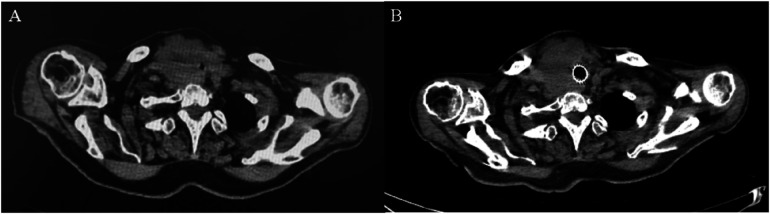
**(A)** on April 7, 2024, the chest CT indicated main airway stenosis. **(B)** On June 30, 2024, the chest CT revealed further infiltration of thyroid cancer compared to previous findings.

**Figure 2:**
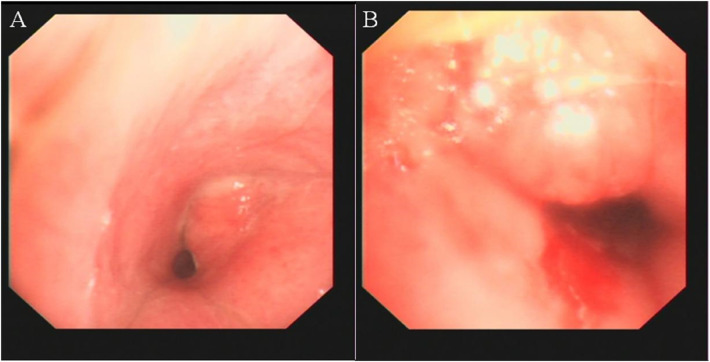
**(A)** Bronchoscopy showed 80% airway obstruction in the patient. **(B)** After rigid bronchoscopic resection, the airway tumor was reduced.

**Figure 3:**
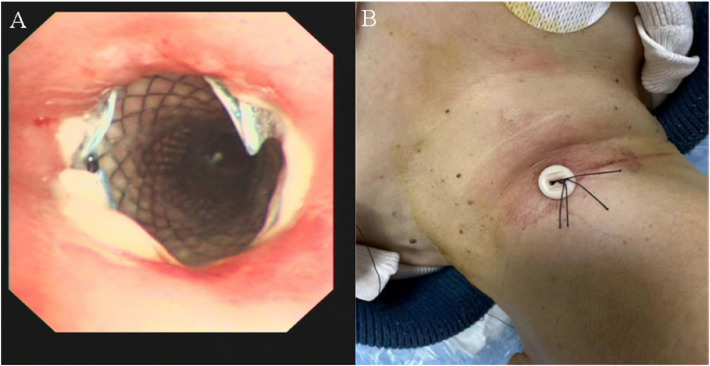
**(A)** Recanalization after stent placement. **(B)** Airway stents secured with a butt on for external fixation.

**Figure 4:**
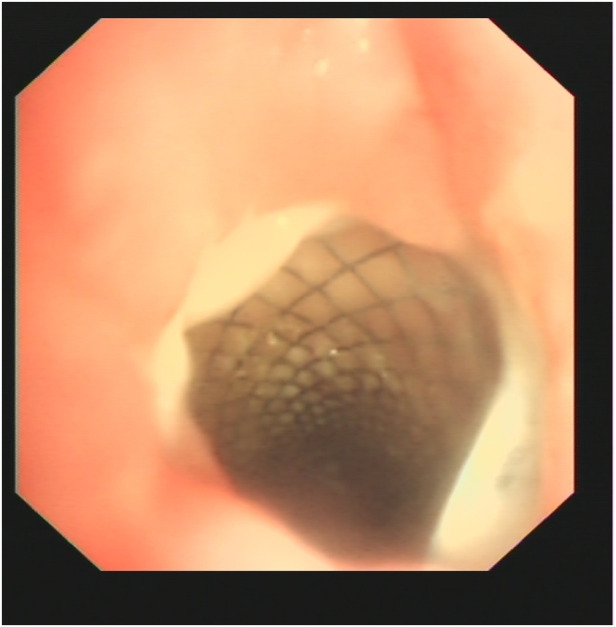
On June 30, 2024, tumor infiltration and granuloma formation around the stent.

## Discussion

Thyroid cancer is a common malignant tumor in the endocrine system, and as the ninth most common cancer in the world, its incidence is high, especially among women, accounting for 75% of all thyroid patients (13). Between 1990 and 2019, the incidence of thyroid cancer in china nearly tripled, an increase of 289.6% (14). Patients with thyroid cancer involving the airway have a higher mortality rate, and those receiving palliative treatment have a 5-year survival rate of less than 40% (15). Traditionally, it is generally accepted that removal of the thyroid gland in the absence of extensive metastasis of the malignant tumor is effective in controlling the disease and achieving long-term remission. When thyroid cancer diffuses into the airway, resulting in central airway stenosis of more than 80%, surgery may not be possible, and tracheotomy, t-tube, or airway stenting may be chosen instead (16). In patients with thyroid cancer invading the airway, the tracheal tissue becomes fragile due to tumor infiltration and impaired blood supply. Following surgery, serious complications such as tracheal stump necrosis and anastomotic leakage are common, often making primary anastomotic reconstruction unsafe (17). In such cases, it is difficult to guarantee negative surgical margins, and the high rate of positive margins increases the risk of distant metastasis. The patient in this case is nearly 80 years old. With the deterioration of physiological function and the decline of cardiopulmonary reserves, the risk of the perioperative risk increases significantly, and the risk-benefit ratio of surgery tends to be unfavorable. At the same time, the patient and his family clearly refused surgical treatment. Therefore, bronchoscopic airway stent placement may represent a reasonable and effective alternative for maintaining airway patency in such patients. Research has shown that bronchoscopic airway stent implantation, can rapidly relieve dyspnea after airway stent placement in 70%–100% of patients (5), with a reduced complication rate and a favorable prognosis. A study showed that the overall remission rate of the treatment group applying airway stent placement reached 87.0%, which was significantly higher than that of the control group with conventional surgical treatment, whereas the incidence of complications was also reduced from 47.8% in the control group to 17.4% in the treatment group (6). The continuous innovation of bronchoscopic interventional techniques and airway stent types leads to a significant reduction in stent-related complications, effectively prolonging the survival years of patients.

According to material composition, existing airway stents can be divided into three categories: metal, silicone, and other hybrid materials. Currently, stents made of silicone or self-expanding metallic stents are most commonly used for central airway stenosis. Silicone airway stents have certain structural limitations, and the adaptability to airway narrowing with irregular morphology and different pipe diameters is poor; while metallic airway stents have better conformability and radial support. Compared with silicone airway stents, metallic airway stents are more suitable for malignant central airway obstruction, as well as airway obstruction secondary to exogenous compression such as mediastinal swelling and fibrosis. Its inner and outer diameter ratio is more ideal, and it is not easy to cause stent migration. In bronchoscopic interventional treatment, it is easier to place the metallic airway stent through the flexible bronchoscope under the guidance of perspective, which is more suitable for patients with dyspnea in emergency scenarios. This type of stent provides structural support to the airway wall, effectively resists external compression, and maintains luminal patency (18). There are currently two types of metal stents: bare metal stents and covered metal stents. Bare metallic airway stents are associated with a higher incidence of granulation tissue formation due to mechanical irritation of the airway mucosa, and their cost may also limit widespread use in some settings. Compared with uncovered metallic airway stents, those covered with a membrane exert less stress on the trachea, maintain firm contact, effectively maintain tracheal patency, reduce airway irritation, and prevent granulation tissue formation or tumor ingrowth, thereby facilitating mucosal healing and airway management (7–9).

In this case report, the patient was in critical condition, with unstable vital signs and poor general status, making her unsuitable for invasive surgical procedures. Due to tumor progression, the thyroid is obviously enlarged, and the space for surgical operation in the anterior neck was limited. Clinically, conventional tracheostomy mostly selects the level of the 2nd to 4th tracheal cartilage ring. The stenosis of this patient is located about 3 cm below the glottic, which is a high-level cervical airway lesion. This anatomical position significantly increases the difficulty of tracheotomy. Tracheotomy placement is prone to bleeding and asphyxiation, and the intraoperative risks are high. In addition, a tracheostomy will increase the local exposure area, and there is also a risk of infection in the subsequent home care process (19). The Montgomery T tube is mainly suitable for limited tracheal lesions. For patients whose neck tumors grow extensively or involve the laryngeal tracheal junction area, its application has certain limitations (20). Previous studies have shown that although some patients with glottic stenosis can receive T-tube treatment, the incidence of postoperative secretion retention is relatively high (21). The clinical effect of the T-tube depends to a large extent on the patient's ability to spontaneously expel phlegm.

Excluding the feasibility of tracheotomy and T-tube placement, inserting a straight covered self-expanding metallic stent was considered the most appropriate option. After the operation, the patient experienced increased wheezing and dyspnea; the stent migration was considered to be triggered by severe coughing. Stent migration, which is the most common complication after airway stent placement, may be caused by respiratory movements, coughing, or swallowing (22). Stent migration often leads to recurrence or worsening of tracheal stenosis, and stent repositioning and fixation are needed as soon as possible. The fixation of airway stents can generally be divided into two categories: internal fixation and external fixation. Internal fixation is performed under bronchoscopy, which is difficult and time-consuming. Meanwhile, the stent is fixed to the bronchial wall and skin by sutures, and the suture tension can cause direct damage to the trachea and surrounding tissues. Considering the high age of the patient, the airway foundation is weak. The diffuse growth of the mass in the patient's airway limits the operating space. If the internal fixation method is used, not only is the operation risk high, but also the cough easily causes tissue damage by suture tension, which damages airway tissue repeatedly. Adnan Majid et al. showed that external fixation can be used as an alternative therapy to internal fixation in some patients who are not candidates for tracheotomy and reconstruction. Multiple case reports evaluated the efficacy and safety of external fixation of airway stents for the treatment of upper-segment tracheal stenosis, and the results showed that this technique significantly reduced the incidence of stent migration and improved overall patient outcomes (10–12, 16). In this case, after the patient's airway stent was displaced, there was obvious respiratory distress, and it was urgent to establish a stable airway. The special devices reported in previous literature for external fixation of the bracket, such as the Bodkin threader, Endo Close device, and Berci needle, may not be equipped under conventional conditions. The center adopts a simple and feasible alternative plan under the limited equipment and materials. Specifically, after strict disinfection, we choose ordinary clothes buttons as external fixing auxiliary devices. In order to improve intraoperative puncture efficiency, we carried out positioning under ultrasound guidance. Using ultrasound guidance can accurately identify the cricothyroid membrane and avoid vascular injury, thereby improving the safety of the procedure. Puncture the cricothyroid ligament and pass through the airway bracket mesh through the 3–0 non-absorbable nylon suture to fix the bracket on the skin and soft tissue in front of the neck. Buttons are used to distribute tension and enhance the fixing effect, thus reducing the risk of stent migration. The potential damage to the airway and surrounding tissues from suture tension was effectively mitigated. The materials used were readily available, reducing the financial burden on the patient. This technique minimized damage while ensuring enhanced stent stability for the patient. Postoperatively, the stent was well fixed, with no active bleeding and normal drainage of airway secretions. April 15, 2024, Electron bronchoscopy showed satisfactory fixation of the airway stent with no signs of migration. Compared with the pre-operation, the patient's respiratory distress disappeared, oxygen saturation improved significantly, and vital signs were stable. During the follow-up process, the patient was admitted to the hospital again due to repeated recurrence of dyspnea. Under the bronchoscopy, further tumor infiltration, secretion retention, and granulation tissue formation could be seen, suggesting that there were certain limitations in airway stent treatment. The cause of frequent dyspnea may be closely related to the rapid progression of the tumor and the decline of the patient's ability to expel phlegm. Therefore, for patients with advanced malignant airway stenosis, airway stents are more of a palliative treatment. External fixation may improve the short-term stability of stents, and its main purpose is to immediately relieve respiratory distress symptoms and improve the patient's quality of life in the short term, but it is difficult to change the natural course of the disease. The lack of long-term follow-up data is one of the main limitations of this case.

## Conclusion

This case suggests that for airway stenosis caused by malignant thyroid tumors invading the trachea, more stable and supportive metallic stents may be selected according to the airway condition and disease progression. External fixation using a simple button-based technique appeared to improve short-term stent stability and reduce the risk of migration. In settings where dedicated devices are unavailable, this approach offers a simple way to stabilize the airway, relieve respiratory distress, and improve short-term symptoms. It is important to recognize that the placement of airway stents can only be used as a palliative treatment, and sequelae such as secretion retention and granuloma formation cannot be avoided in the later stage, nor can they stop the further progression of the tumor. The follow-up period of this study was limited to two months, providing only preliminary evidence for the role of external fixation in improving the stent stability. In the future, it is necessary to carry out research with larger cohorts and extended follow-up to evaluate the safety and durability of this technique.

## Data Availability

The original contributions presented in the study are included in the article/Supplementary Material, further inquiries can be directed to the corresponding author.
